# Family Support and Hope among People with Substance Use Disorder in China: A Moderated Mediation Model

**DOI:** 10.3390/ijerph19169786

**Published:** 2022-08-09

**Authors:** Wenqiang Cai, Yijie Wang

**Affiliations:** 1 School of Public Administration, Hohai University, Nanjing 211100, China; 2 Department of Public Security Management, Jiangsu Police Institute, Nanjing 210031, China

**Keywords:** family support, resilience, hope, substance use disorder, relapse

## Abstract

Studies have shown that hope is an important protective factor. At present, few of the available studies on hope have been conducted on people undergoing compulsory rehabilitation. This study explores the mediating role of resilience between family support and hope, and whether relapse plays a moderating role between family support, resilience, and hope. A total of 647 people with substance use disorder completed surveys on Perceived Social Support from Family Scale, Connor-Davidson Resilience Scale, and Herth Hope Index. Structural equation modeling was used to examine the moderated mediation analysis. Family support not only has a direct effect on hope, but also has a significant indirect effect on hope through resilience. The indirect effect of family support on hope via resilience was significant among both the non-relapse group and relapse group; in addition, both the association between family support and resilience and the relation between resilience and hope were moderated by relapse experience. The results indicate that interventions targeting resilience might be an effective approach to improving hope among people with substance use disorder in China.

## 1. Introduction

The international nature of; the drug problem is unprecedented, affecting the development of the community of a shared future for mankind in an interwoven way. According to the World Drug Report 2021 [1], around 275 million people have used drugs in the past year, of whom approximately 36.3 million were estimated to suffer from drug use disorders. Furthermore, COVID-19 has brought about many new changes in the global production, supply, trafficking patterns, and consumer demand for drugs, increasing the challenges of addiction treatment and rehabilitation. China’s drug problems are also prominent. In 2020, about 1.801 million people have used drugs, 155,000 newly discovered people who use drugs, and 272,000 people recurrence of use [2].

As one of the main bodies of substance use disorder control, people with substance use disorder (PWSUD) are a combination of “Victims”, “Violators”, and “Patients”, and their physical and mental health status is not optimistic. At the same time, they are more likely to appear in public as illegal or social outliers and deeply suffer from this “labeling”. As a drug rehabilitation model in China, compulsory isolation for drug rehabilitation relies mainly on the top-down intervention of the state’s administrative power, often ignoring the subjectivity and initiative of PWSUD [3]. Therefore, it is crucial to reinforce the positive psychological factors that shape the physical and mental health of those in compulsory isolation who need to prioritize their health for recovery. Through qualitative research, Gao et al. [4] pointed out that the change in PWSUD’ cognition, especially the change of implicit cognition, is the breakthrough point of psychological correction. A growing body of research shows that hope in cognition is an important factor in disease rehabilitation and has a positive effect on health [5,6]. As the main detoxification model in China, compulsory isolation detoxification is different from mainstream Western detoxification. Moreover, due to COVID-19, all compulsory isolation and rehabilitation institutions (CIRI) strictly implement epidemic prevention regulations. To protect the health of staff and PWSUD in CIRI, visits to PWSUD by persons, including immediate family members, were temporarily banned. There is no unified conclusion about how this isolation and temporary ban affects psychological and family support. Although much attention has been paid to the element of “hope” in academic research on drug rehabilitation, little research has been conducted about hope in PWSUD.

### 1.1. Hope

Hope has received much attention from the academic community as an important concern in positive psychology research. However, no universal definition of hope exists. It is seen as a future-oriented psychological power that inspires new values and encourages individuals to change their status quo [7,8]. Hope has also been conceptualized as a psychological state [9]. Based on the research of Dufault and Martocchio [10], Herth [11] described hope as “a multidimensional dynamic life force characterized by a confident yet uncertain expectation of achieving good, which, to the hoping person, is realistically possible and personally significant”.

In research on drug rehabilitation, numerous studies have shown that hope is a key characteristic of recovery [12]. It plays a protective role in recovery from withdrawal because it prevents many behavioral and mental problems in life [13,14]. As Moulden and Marshall [15] suggest, real hope is the motivation to overcome obstacles such as physical and mental illness; it is the starting point of the rehabilitation process and the basis of a very important determination to achieve any goal [16]. Hope maybe help patients conceptualize their aspirations for recovery, coping, and adaptation [17], higher hope can increase the motivation of patients to participate in the recovery process [18]. At the same time, there is an important relationship between hope and chemical substance use and recovery from chemical substance use disorder [12]. Research by Mathis et al. (2009) on hope in substance abuse rehabilitation confirms that hope is an important resource to help individuals overcome the challenges of rehabilitation [19]. In addition, hope has been incorporated as a predictor in family therapy to address addiction and promote rehabilitation [20].

In this context, hope is not self-centeredness, but comes from communicating with others and interacting with groups. It is both, the expectation of change in relationships with others and the expectation of being accepted and respected by others [21]. Theoretically, family support appears to be associated with higher levels of hope. Obayuwana and Carter [22] asserted that perceived family support is one source of hope. In extending hope theory, Bernardo [23] stated that hope is the result of the joint action of internal and external hope trajectories. The internal hope trajectory refers to the motivation for individuals to realize cognition as a goal. External hope trajectories include family, friends, and spiritual power, which may increase the degree of hope. Although many studies have examined the role of hope and related factors, there are few studies on hope in PWSUD, particularly as the antecedent and influence mechanisms of their hope are unclear.

### 1.2. Family Support and Hope

Detoxification is a very challenging and arduous process that requires not only having a strong body but also a strong will and mentality to resist persistent physical or psychological addiction, which also causes significant physical and psychological pressure. There are many ways to deal with this, and one of the most important ways is through social support. Social support helps individuals feel loved, valued, and respected [24], and effective family support can help individuals stay healthy, get help, reshape their confidence, and integrate into social groups [25]. Social support has been found to provide valuable resources for coping with stress and maintaining good health across cultures and groups [26]. It is a multidimensional concept, with family support being one of the most important dimensions, including instrumental support for material help, informational support for advice or guidance, and emotional support for compassion, care, trust, and comfort [27]. Family support is especially important for a recovering substance use disorder as the inner strength to overcome the challenges of addiction is not so easily acquired [28]. As a result, family support is considered to have a positive impact on mental and physical health [29].

Many studies have shown a significant positive correlation between family support and hope [30,31]. One of the roles of the family, which can be a source of social support for other family members to address their problems, is to assist PWSUD to recover from withdrawal [32]. To ensure successful recovery for PWSUD, family support must not only be present throughout the process of recovery from withdrawal but also afterwards to help prepare them for a new phase of life. Therefore, family involvement in addiction treatment is beneficial for the rehabilitation of PWSUD [33], as support from the family can provide them with the confidence to recover from substance use disorder [32], relieve the stress of recovery, prevent anxiety, give strength and motivation to persevere, and raise the individual’s hope and confidence in the future [34,35]. Conversely, a lack of family support can lead to a lack of life goals and hope, and increased dependence on drugs [28].

### 1.3. The Mediating Role of Resilience

Resilience is a complex construct that refers to the ability to recover and adapt satisfactorily to threats, difficulties, and stress [36], enabling individuals to actively rebuild their lives [37]. It is a dynamic process that occurs as a personality trait throughout life [38], maintaining physical and mental health by mitigating the negative consequences of adversity [39]. As highlighted by the positive relationship between resilience and hope, it is strongly associated with an individual’s mental ability. A study on resilience, hope, and social support among caregivers showed that they correlated with each other [40]. Moreover, research has found that positive emotional experiences can effectively promote the recovery of individuals with high resilience from daily stress [41]. Positive emotions may stem from and contribute to building resilience [42]. For example, there is a direct relationship between resilience and hope, which has been widely demonstrated [43]. Wu [44] found that resilience had a significant positive effect on hope after studying a family member who had been traumatized by crime.

Resilience involves not only individual mental abilities or traits but also mutually beneficial relationships between individuals and their environment [45], such as individuals’ positive access to and use of external resources [46]. It enables individuals to cope with their situations and changes in their lives in a dynamic process influenced by life events and challenges, in which social support also plays an important role [47]. Family support is positively related to resilience [48]. Research has found that optimism and self-efficacy, and family and social support as protective factors can promote the development of resilience in children from addicted families [49]. Although resilience may be impaired by some risk factors, it may also help adolescents cope better with stressful events in their lives [50]. Based on these correlations, this study hypothesizes that resilience serves as a potential mediator in the relationship between family support and hope. However, few studies have examined this mediation model.

### 1.4. The Moderating Role of Relapse Experience

Although family support may have a direct impact on hope through resilience, not all people with lower resilience have lower hope. Therefore, it is necessary to explore the influential factors that may strengthen or weaken the link between family support, resilience, and hope. This study examined a hypothesis that the family support–resilience link and the resilience–hope link in the indirect association between family support and hope would be moderated by relapse experience. Relapse is a common problem in drug rehabilitation [51]. PWSUD who lack social support after returning to society may choose to relapse [52]. The social support of family, relatives, and friends can not only create supervision but also enhance their sense of family responsibility, thus strengthening their resilience and belief in addiction treatment. Moreover, research on the relapse resiliency model addresses how social support is an important factor in building resiliency. Individuals within a system choose how to react to the pain, either through a compulsive cycle of relapse (lack of resilience) or through a coping cycle of recovery to maintain resiliency. Relapse resiliency is implemented in the recovery phase in the process model of addiction and recovery as a way to maintain lifelong resiliency to relapse [53].

Gorski [54] argued that relapse is a complex process that can lead to recovery from substance use or emotional and psychological problems over time. For example, several studies have found that people with recurrent substance use disorders tend to have a low sense of self-efficacy, which leads to psychological problems by undermining resilience [55]. A study showed that people with low mental toughness were at an increased risk of relapse, indicating that recovery support programs are needed to enhance their mental toughness to prevent the same. The more relapse experience, the lower the level of resilience [56].

### 1.5. Current Study

In conclusion, there are many studies on the above variables, most of which focus on the western countries’ own detoxification model, while there are relatively few studies on compulsory detoxification in China. Meanwhile, the relationship between family support and hope, as well as the possible mediating and moderating role, remains to be explored. It is very important to determine the supportive psychological factors in the process of PSWUD detoxification in order to help patients recover smoothly. Based on the existing studies on the relationship between family support, resilience, and hope, the purpose of this study was to evaluate the mediating role of resilience between family support and hope and to explore whether relapse experience plays a moderating role in this relationship. The following two hypotheses will be tested: (1) whether resilience mediates the relationship between family support and hope, and (2) whether relapse experience would moderate the indirect association between family support and hope via resilience.

## 2. Methods

### Participants and Procedure

From May to July 2021, we used the cluster random sampling method to select six CIRI in Jiangsu Province, China, to conduct the survey. To ensure the smooth conduct of the survey, the education cadres were commissioned to explain the purpose of the study to the participants and introduce the questionnaire before the survey was conducted. A total of 751 questionnaires were distributed, and 647 valid questionnaires were returned. Table 1 shows the demographic characteristics of the participants.

Prior to the formal investigation, we conducted a questionnaire survey to verify the validity of the questionnaire. In Nanjing CIRI, 20 participants were selected to voluntarily conduct a questionnaire survey. At the same time, the education cadres were invited to check the content of the questionnaire. The questionnaire was revised to remove some language that was considered sensitive and to modify some of the words and situations to make the questionnaire more relevant to the actual situation of PWSUD. All survey procedures were approved by the Ethics Committee of Hohai University. All participants had given their informed consent prior to the start of the investigation and could withdraw at any time during the course of the investigation.

## 3. Measures

### 3.1. Perceived Social Support from Family Scale

The Perceived Social Support from Family Scale (PSS-Fa) was compiled by Procidano et al. (1983) and revised by Zhang et al. (2001) [57,58]. There were 15 items on the scale, which were scored by two points, namely “1 = Yes” and “0 = No”; higher scores indicated higher family support. Studies have shown that the scale has high reliability and validity [59], and the Cronbach’s α in this study was 0.767. Confirmatory factor analysis (CFA) indicated that the model fit was acceptable: χ^2^/DF = 2.40, CFI = 0.96, TLI = 0.94, and RMSEA = 0.05.

### 3.2. Connor-Davidson Resilience Scale

The Connor-Davidson Resilience Scale (CD-RISC) was developed by Connor and Davidson [60] and revised by Yu et al. (2007) [61]. The CD-RISC is a 25-item scale designed to measure three components of resilience: Tenacity, Self-improvement, and Optimism. On a Likert scale of 5, ranging from “1 = never” to “5 = always” with higher scores indicating greater resilience. The results have shown that the Chinese version of the scale has good reliability and validity and is suitable for the Chinese population [61]. In this study, Cronbach’s α was 0.938, and Cronbach’s α of the three dimensions were 0.912, 0.797, and 0.709, respectively. CFA indicated that the model fit was acceptable: χ^2^/DF = 3.36, CFI = 0.92, TLI = 0.91, and RMSEA = 0.06.

### 3.3. Herth Hope Index

The Herth Hope Index (HHI) was originally compiled by Herth [5], and the Chinese version of the HHI was translated by Zhao et al. (2000) [62]. The scale has 12 items and is based on three dimensions: cognitive-temporal (T), affective-behavioral (P), and affiliative-contextual (I). Participants were asked to rate each item on a four-point Likert scale, ranging from 1 to 4 (1 = strongly disagree, 2 = disagree, 3 = agree, 4 = strongly agree), with higher scores indicating higher levels of hope. The scale has been shown to have high reliability and validity [63]. In this study, the Cronbach’s α of the scale was 0.883, and the Cronbach’s α of the three dimensions was 0.640, 0.781, and 0.667, respectively. CFA showed that the model fit was acceptable: χ^2^/DF = 2.62, CFI = 0.97, TLI = 0.96, and RMSEA = 0.05.

### 3.4. Moderating Variable

We use “how many times have you entered a rehabilitation institution for treatment?” to measure relapse experience. The options to this question are 1, 2, 3, 4, 5, or above. The “1” represents initial entry into the rehabilitation institution, it was coded such that “0 = non-relapse”, and the “2 or more” was coded “1 = relapse”.

### 3.5. Statistical Analysis

First, a common method bias test was conducted using Harman’s single-factor test with a common factor number of 1; all items such as resilience, family support, and hope were subjected to factor analysis, and CFA was conducted using Amos 25.0. Second, the results were subjected to descriptive tests, difference analysis, and correlation analysis. SPSS 19.0 independent sample *t*-test was used to test for significant differences in family support, resilience, and hope in terms of whether or not they relapsed, and Pearson’s product–moment correlation was performed. Third, this study examined the mediating role of resilience between family support and hope through mediation analysis. Fourth, this study examined whether the mediation process was moderated by relapse. Moderated mediation is usually used to test whether the size of the mediation effect depends on the value of the moderating variable [64]. In addition, we used the bootstrap method to test the significance of all effects and obtained the standard error of the parameter estimation. The bootstrap method generates 95% deviation-corrected confidence intervals (95% CI) for these effects from 2000 resamples, with confidence intervals that do not contain 0 indicating significant effects [65].

## 4. Results

### 4.1. Common Method Bias

Harman’s single-factor test [66] was used to analyze all items on the CD-RISC, PSS-Fa, and HHI. The results showed that there were nine factors with characteristic roots greater than one, and the variance explained by the first factor was 25.719%, which is less than the critical value of 40%. At the same time, CFA was performed using Amos 25.0. The results showed that χ^2^/DF = 5.95, NFI = 0.52, CFI = 0.57, GFI = 0.56, TLI = 0.55, RMSEA = 0.09, and the model fit was poor. Therefore, there was no serious problem with common method bias in this study.

### 4.2. Preliminary Analysis

There were no significant differences in hope associated with gender, age, years of first drug abuse, marital status, and education, either in relapse or non-relapse. An independent samples *t*-test was conducted on family support, resilience, and hope. The results showed that there were significant differences in resilience (*p* < 0.05) and hope (*p* < 0.001), wherein the scores of resilience and hope of the non-relapse group were significantly higher than those of the relapse group, and the scores of family support were not significantly different. The results are shown in Table 2. Pearson’s product-moment correlation was used to analyze the family support, resilience, and hope in non-relapsers and relapsers, and correlations of all the variables are presented in Table 3 The results showed that family support, resilience, and hope were significantly correlated (all *p* < 0.001). These bivariate correlations support the next mediation analysis.

### 4.3. Testing for Mediation Effect

**Figure 1:**
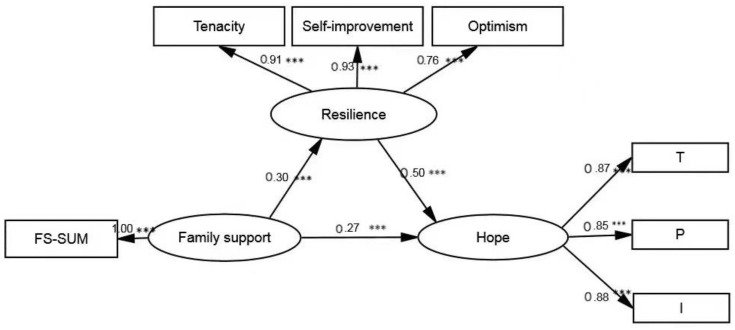
Results of the mediation analyses in PWSUD. *** *p* < 0.001.

Structural equation modeling (SEM) was used to examine the mediating effect of resilience, and the fit between the model and the data was examined by maximum likelihood [67]. The results in Table 4 showed that the model fits well. Family support can not only positively predict hope but also indirectly influence hope through resilience. In other words, resilience partially mediated the relationship between family support and hope. The mediating effect value was 0.15, the 95% CI was [0.11, 0.20], and the proportion of the total effect was 35.71%. Thus, Hypothesis 1 is verified. The detailed path model is shown in Figure 1.

### 4.4. Testing for Moderated Mediation

To explore the moderating effects of relapse experience in the resilience–hope link in the indirect association between family support and hope, this study further used a multi-group analysis to compare the relationship between the relapse and non-relapse groups, and developed an SEM between family support, resilience, and hope using Amos 25.0. In the multi-group analysis, a non-restrictive model (allowing the path coefficient to be freely estimated between the relapsed and non-relapsed groups) was compared with a restrictive model (limiting the path coefficients to be equal in the relapsed and non-relapsed groups). The acceptability of the model was determined based on the significance of Δχ^2^ and ΔDF [68]. It was found that when the limiting path coefficient was equal in the relapse and non-relapse groups, the change value of the fit index of the model was very small. The *p*-value of Δχ^2^ and ΔDF was 0.611, which did not reach the significant level of 0.05, as shown in Table 5. In other words, the characteristics of the non-restrictive model and the restrictive model can be regarded as the same, whether or not the relapse has an overall moderating effect. Typically, researchers would likely suggest that if models are essentially the same, then the “smaller”, nested model (the model with fewer parameters to be estimated and therefore more degrees of freedom) might be chosen as a representative model for the sake of parsimony and computation cost [68]. Thus, the following discussion uses the path coefficients of the restrictive model.

**Figure 2:**
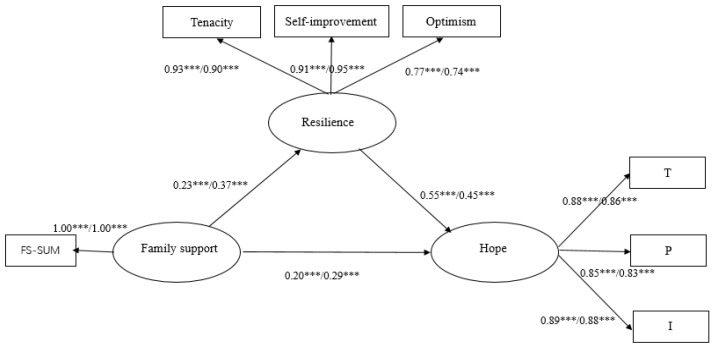
A multi-group analysis model of resilience of PWSUD between family support and hope. Note: The first data is the standard coefficient for the non-relapsers, and the second data is the standard coefficient for the relapsers. *** *p* < 0.001.

Family support can not only positively predict hope but also indirectly influence hope through resilience, which has a significant mediating effect on family support and hope relationships in the non-relapse group, with a mediating effect value of 0.13 and the 95% CI [0.07, 0.20], accounting for 39.39% of the total effect. Meanwhile, in the relapse group, family support not only positively predicted hope but also indirectly influenced hope through resilience, which had a significant mediating effect on family support and hope relationships, with a median effect of 0.17, 95% CI [0.11, 0.23], and a 36.96% share of the total effect. As shown in Figure 2, the effect of family support on resilience was stronger for the relapse group (*β* = 0.37, *p* < 0.001) than for the non-relapse group (*β* = 0.23, *p* < 0.001), but the effect of resilience on hope was lower for the relapse group (*β* = 0.45, *p* < 0.001) than for the non-relapse group (*β* = 0.55, *p* < 0.001).

## 5. Discussion

### 5.1. The Mediating Role of Resilience

The results show that resilience is a mediator variable between family support and hope. Family support not only directly influences hope but also influences hope via resilience. This is consistent with previous research [30,44]. Family support positively affects hope, that is, the higher the level of family support, the higher the level of hope. When a person in compulsory isolation faces family conflicts and low levels of family support, it can lead them to believe that no one wants to accept them, a situation that can easily cause them stress and problems such as pessimistic psychology (such as depression and despair) and social adjustment difficulties, among others [28]. Individuals with higher levels of family support who face compulsory isolation for drug rehabilitation may be more inclined to believe that they can gain understanding and help from their families, strengthening their belief that they can get rid of addiction, which further promotes the development of resilience [35]. In family culture-oriented China, the role of the family in drug rehabilitation is considered more important than in individualistic Western cultures, especially with support from blood-related parents, children, siblings, and couples. Family support is seen as an important factor in encouraging PWSUD to start and continue treatment [69]. Therefore, the level of family support for them needs to be raised because, whether in CIRI for drug rehabilitation or at the end of the isolation program for social rehabilitation, they will feel accepted by their families and receive emotional and material support, and path advice they need to recover from family support.

### 5.2. Analysis of the Moderating Role of Relapse Experience

The results also reveal relapse experience moderates the relationship between family support, resilience, and hope. Specifically, regardless of whether they relapsed, family support not only predicted hope but also indirectly influenced hope through resilience. However, compared with the relapse group, the mediating effect of resilience between family support and hope was lower in the non-relapse group. In addition, both the association between family support and resilience and the relation between resilience and hope were moderated by relapse experience.

According to the relapse resilient systemic process model of addiction and recovery, individuals within a system choose how to react to the pain (e.g., physical matter, mental matter, emotional matter, social matter), and many individuals rely on relapse to reduce their discomfort [53]. However, the guilt or shame that individuals experience as a result of relapse produces more pain, which further reduces resilience. Therefore, the relapse group needs more family support to strengthen resilience, in order to enhance their belief and hope of recovery. Specifically, compared with the non-relapse group, family support of the relapse group has a greater significant impact on their resilience, while resilience has a smaller significant impact on their hope. However, the reality is that frequent relapse leads to deterioration of social support, including family tensions, interpersonal conflicts, and greater social prejudice, discrimination, isolation, and distrust [70], as well as a “lifetime addict” label phenomenon and stigma [71]. Compared to healthy populations, relapsers experience more frequent interpersonal conflicts and are more likely to perceive negative support. In one study, PWSUD expressed the need for more family support to facilitate effective recovery. Unfortunately, most of the immediate family members of PWSUD provided little or no support, and family relationships deteriorated further when family members became aware of substance abuse problems.

During COVID-19, communication between PWSUD and their families was temporarily blocked due to the need for preventive measures to mitigate the spread of the disease, and the temporary ban on visits to CIRI somewhat weakened family ties and family support. When PWSUD face deteriorating family relations or even family conflicts, as well as social prejudice or stigmatization, these can lead to a stressful and challenging future in terms of rehabilitation, life, and work. They are more emotional, paranoid, autistic, depressed, and have higher levels of shame than the average person [72], and may be more vulnerable to adverse psychological effects [73], especially for those who suffer from mental health problems before forced isolation and social alienation. According to the resource consumption theory of self-control [74], tasks that require self-control deplete self-control resources. These may be depleted in the management of negative emotions, such as coping with depression and shame, which can impede subsequent self-control and lead to reduced resilience. When PWSUD are in low family support and low resilience situations, they will feel confused, with low hope, or even hopelessness for the future. To escape this situation, individuals often resort to relapse to respond negatively.

### 5.3. Implications and Limitations

The results of this study suggest that CIRI should pay special attention to PWSUD from a low family support level, who are more likely to have low hope problems due to low family support levels, and then lead to relapse. In terms of communication skills, especially in special periods, CIRI should make active use of modern communication technology and try “Cloud” communication such as remote video visits based on voice calls, to strengthen emotional ties between family members and PWSUD. In addition, group counseling and psychological consultation can be carried out to enhance positive psychological qualities, such as optimism, self-efficacy, and resilience, to help the PWSUD recover quickly and positively from the imbalance, and look forward to the future hopefully.

This study has several limitations that must be noted. First, the current study focused on PWSUD, which is a special part of the drug rehabilitation group, and it is unknown whether the findings of the study can be applied to other types of substance use disorders or other characteristic groups, and further research is needed. Second, there was a self-report bias. This study used a questionnaire method, which was conducted in CIRI and filled out by the participants themselves. The environment in which surveys were taken may have limited participant willingness to provide unfiltered and accurate responses. Third, the study focused on a cross-section of time that more closely reflected what the subjects were exposed to at that time. However, with the development of drug rehabilitation and changes in the surrounding environment, many of their conditions may change, such as those who went into the CIRI before the COVID-19 epidemic, and their hopes and family support may be higher than those who entered the CIRI after the COVID-19 epidemic, which will require further study in the future.

## 6. Conclusions

Based on the analysis and discussion, family support of PSWUD directly affects hope, while resilience has a mediating effect. Furthermore, relapse experience had a significant moderating effect. Compared with the relapse group, the mediation effect of resilience in family support and hope relationships is lower in the non-relapse group. The results of this study suggest that family members should actively support the withdrawal and rehabilitation of PWSUD, and rehabilitation centers should take a variety of measures to improve their resilience to ignite their hopes.

## Figures and Tables

**Table 1:** Demographic characteristic of the participants (n = 647).

Items	Values	Mean ± SD, (Range)
**Number of participants**	647	
**Gender (% male)**	78.8	
**Age (years), (Range)**		38.58 ± 8.44, (22–68)
**Age of first drug use (years), (Range)**		25.85 ± 7.58, (13–52)
**Education level (%)**		
Junior high school or below	63.4	
Secondary	27.8	
College or above	8.8	
**Marital status (%)**		
Married/cohabiting	46.1	
Separated/widow/divorced	30.3	
Single	23.6	

**Table 2:** The *t*-test of family support, resilience, and hope of non-relapsers and relapsers.

	Non-Relapsers (N = 288)	Relapsers (N = 359)	*t*
Family support	0.704 ± 0.223	0.698 ± 0.202	0.390
Resilience	3.425 ± 0.672	3.320 ± 0.658	1.988 *
Hope	3.248 ± 0.492	3.119 ± 0.495	3.296 ***

*** *p* < 0.001; * *p* < 0.05.

**Table 3:** Correlation analysis of family support, resilience, and hope for relapsers and non-relapsers.

	Family Support	Resilience	Hope	M	SD
Family support	—	0.340 ***	0.423 ***	0.698	0.202
Resilience	0.227 ***	—	0.489 ***	3.320	0.658
Hope	0.307 ***	0.544 ***	—	3.119	0.495
M	0.704	3.425	3.248	—	
SD	0.223	0.672	0.492		—

Above “—” is the correlation coefficient among the variables of relapsers, and below “—” is the correlation coefficient between the variables of non-relapsers. *** *p* < 0.001.

**Table 4:** The model fitting index of the influence of family support and resilience on the hope.

	χ^2^/df	NFI	CFI	GFI	TLI	RMSEA
Mediating effect model	1.51	0.99	0.99	0.99	0.99	0.03

**Table 5:** Multi-group SEM fitting index.

Model	χ^2^	df	χ^2^/df	CFI	TLI	Δχ^2^	ΔDF	*p*
Unrestricted	38.069	20	1.903	0.994	0.987	6.687	3	0.083
Restricted	44.756	23	1.946	0.992	0.990			

## Data Availability

The data presented in this study are available on request from the corresponding author.
